# Insight into the Feasibility and Acceptability of a Multi-lifestyle Dementia Risk Intervention

**DOI:** 10.1093/geroni/igab046.3501

**Published:** 2021-12-17

**Authors:** Laura Dodds, Joyce Siette

**Affiliations:** Macquarie University, Macquarie Park, New South Wales, Australia

## Abstract

Lifestyle interventions based on behaviour change principles may provide a useful mechanism in reducing dementia risk amongst older adults, however intervention acceptability remains relatively unexplored. We assessed the feasibility and acceptability of BRAIN BOOTCAMP, an Australian initiative aiming to improve dementia literary and reduce dementia risk by delivering a brain health box addressing multiple lifestyle factors through education, physical prompts and an individualised brain health profile. Semi-structured phone interviews were conducted with participants (N=94) at completion of the program (3-months) using a theoretical sampling approach to select a range of participants with varying brain health scores, age, gender, education and locality. Interview topics included participants’ overall experience and suggestions for program improvement. Interviews were transcribed and analysed using thematic analysis. Participants were mostly female (79%), with a mean age of 72.6 years (SD=5.4), from an English-speaking background (89.4%) and resided in metropolitan areas (76.6%). Participants positively perceived the program, resulting in high usability and acceptability. Valuable aspects included building dementia awareness in an innovative way, and having re-assessments which identified areas for personal improvement. Participants further discussed how the program prompted lifestyle change, including setting goals (e.g., physical activity) and facilitated a general awareness of their brain health. Suggested improvements included shorter surveys, regular check-ins, and specific tailoring of the program to be more inclusive for older adults with varying levels of health. Our study demonstrated that a simple, innovative program could be a promising medium for delivering comprehensive educational resources and induce lifestyle change for older adults.

